# Epigenetics of Urological Cancers

**DOI:** 10.3390/ijms20194775

**Published:** 2019-09-26

**Authors:** Wolfgang A. Schulz, Karina D. Sørensen

**Affiliations:** 1 Department of Urology, Heinrich Heine University, 40225 Düsseldorf, Germany; 2 Department of Clinical Medicine, Aarhus University & Department of Molecular Medicine, Aarhus University Hospital, 8200 Aarhus, Denmark; kdso@clin.au.dk

The major urological cancers comprise prostate adenocarcinoma, urinary bladder (or upper urinary tract) carcinoma, renal cell carcinoma, testicular cancer and penile carcinoma, in this order of incidence, each with various histological and molecular subtypes. Genomic changes in these cancers are becoming comprehensively characterized, e.g., through large-scale genomic sequencing initiatives. Molecular profiling of epigenetic changes in these cancers is also underway.

As originally conceived, the concept of “epigenetics” aimed at explaining how a diversity of distinct cellular phenotypes can be generated from the same genome during embryonic development and tissue homeostasis. We now know that this is achieved by the interplay of various regulatory mechanisms, likewise termed “epigenetic”, which include DNA methylation, histone modifications and chromatin remodeling, which are implemented by a large array of chromatin regulator proteins and non-coding RNAs.

In an analogous manner, epigenetic mechanisms establish the aberrant phenotype of tumor cells. Therefore, elucidating the causes and consequences of altered epigenomes is an essential prerequisite to understanding cancer development and progression. In principle, epigenetic mechanisms may contribute to cancer development and progression in three different ways (Figure 1).

In one extreme scenario, epigenetic mechanisms on their own might cause a cancer in the absence of mutational changes to the genome. Collectively, the interaction of various epigenetic mechanisms could “freeze” precursor cells in a state of proliferation while blocking their differentiation. As Lobo et al. [1] describe in their contribution to the IJMS special issue on the “Epigenetics of Urological Cancers”, this scenario may apply to some germ cell cancers. Most of these cancers in children and younger men are practically devoid of relevant point mutations and contain a limited number of copy number alterations (CNAs). Together, epigenetic mechanisms and CNAs appear to lock the cancers in various states of germ cell development. How this occurs precisely requires further investigation, but it is already clear that the peculiar epigenetic states of germ cell cancers can be exploited to generate specific biomarkers [1] and novel therapeutic approaches [2].

A second scenario applies to cancers that are primarily driven by genomic mutations, i.e., point mutations or numerical/structural chromosomal aberrations. In such cancers, epigenetic mechanisms would be employed to implement the altered phenotype of the cancers. In their contribution to this special issue, Frame and Maitland [3] delineate how epigenetic mechanisms—in the context of a mutated genome—contribute to altered cell lineages and differentiation of prostate carcinoma. Specifically, epigenetic mechanisms, together with additional mutations, allow prostate cancer cells to adapt to anti-androgenic treatment, leading to therapy resistance and tumor relapse. In clear cell renal cell carcinoma, similarly, common mutations in chromatin regulator genes allow the tumor cells to survive in their characteristic non-physiological state of permanent pseudo-hypoxia, thereby complementing the inactivation of the crucial Von_Hippel-Lindau (VHL) tumor suppressor [4].

A critical and usually lethal step in tumor progression is metastasis, which often involves epithelial–mesenchymal transition (EMT) as one crucial step. Being reversible by mesenchymal-epithelial transition (MET), EMT is clearly a regulated process which also occurs during normal development and wound healing. Unsurprisingly, therefore, EMT is directed by epigenetic mechanisms, as detailed for bladder cancer in the contribution by Monteiro-Reis et al. [5].

The third scenario concerns cancers in which the epigenetic state is altered as a direct consequence of mutations in epigenetic regulator genes. In urothelial bladder cancer, mutations in chromatin regulator genes like *KDM6A*, *KMT2C* and *KMT2D* are pervasively found in basically every case [6], suggesting that they are fundamental to the establishment of this cancer type. Unfortunately, it is not yet clear in which ways these mutations drive tumor development. As Monteiro-Reis et al. [5] point out, some of these mutations may also influence EMT and thereby invasion and metastasis.

Although rarely affected by genomic alterations, another group of epigenetic regulators, histone deacetylases (HDACs), are frequently deregulated in urothelial carcinoma. These enzymes are not only of interest for their functions in tumor pathogenesis but also because they provide good targets for therapy. Some of their inhibitors (HDACi) are already employed in the treatment of cancer and other diseases. In this special issue, Giannopoulou et al. [7] review the expression pattern, potential function and therapeutic potential of the overall 18 members of the four HDAC classes in urothelial bladder cancer. Their most important conclusion is that HDACi may be best administered as components of drug combinations in this cancer type. An important addition to this review is the original article by Buckwalter et al. [8] who have systematically investigated the expression of HDACs in cellular and animal model systems of bladder cancer, comparing their results with data from primary tumors. Both articles are complemented by an original investigation addressing the function of HDAC5 [9]. This further supports the conclusion that predominantly class I HDACs, like HDAC1 and HDAC2, drive proliferation and survival of urothelial carcinoma cells [7,10], whereas class IIA enzymes, like HDAC4 and HDAC5, usually impede tumor growth. Intriguingly, however, and in keeping with the ideas outlined by Monteiro-Reis et al. [5], overexpression of HDAC5 induced EMT in one urothelial carcinoma cell line [9].

While HDACi may serve to illustrate the therapeutic application of insights into cancer epigenetics, DNA methylation-based assays have become the paradigm for diagnostic applications. This is due to the high chemical and biological stability of DNA methylation, the availability of a variety of elegant and robust assays, and especially to the often close association of particular DNA methylation changes with specific tumors or even subtypes and stages. Since urological cancers moreover abut on the urinary tract, assays for diagnostic, prognostic or predictive biomarkers may use cells or components from urine in a convenient and non-invasive manner. Despite these many advantages, implementation of methylation-based assays in the clinic is proceeding at a slow pace [11]. In this special issue, reviewing the current state of DNA methylation-based assays using urine for the detection of urological cancers, Larsen et al. analyze in particular the factors underlying this problem and suggest routes to address them [12]. Two original papers on DNA methylation expand the theme. Bjerre et al. [13] describe a new combination of hypermethylated genes assayed by quantitative methylation-specific PCR which could serve as a prognostic biomarker in prostate cancer, identifying patients at risk of recurrence following radical surgery with high sensitivity and specificity. Another prognosis of the clinical course of prostate cancer after surgical intervention might be obtained by measuring the level of 5-hydroxymethyl-cytosine (5hmC), a DNA base generated from the common methylcytosine via oxidation by TET (Ten-Eleven Translocation) dioxygenases. Kristensen et al. [14] report that high levels of this modified base are significantly associated with worse outcomes in the subgroup of patients with ERG (ETS-related gene)-negative tumors, which comprise about half of all cases,.

In conclusion, the reviews and original articles in the IJMS special issue on the “Epigenetics of Urological Cancers” provide an illustration on how progress in this field contributes to our improved understanding of pathogenesis, provides novel targets for therapy and biomarkers for detection and prognosis in this diverse group of cancers. The editors hope that the contributions to this issue will stimulate further research and reveal novel biological insights, but most of all will help to improve prevention, diagnosis and therapy of urological cancers.

## Figures and Tables

**Figure 1:**
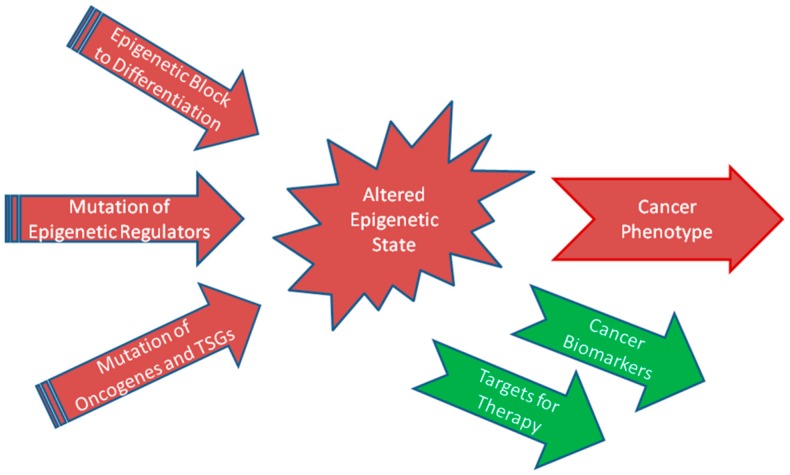
Three factors establishing the altered epigenetic states of (urological) cancers and their consequences. TSGs, tumor suppressor genes.

## Abbreviations

CNA	Copy number alteration
EMT	Epithelial mesenchymal transition
HDAC	Histone deacetylase
HDACi	Histone deacetylase inhibitor
MET	Mesenchymal epithelial transition
